# Small Caliber Compliant Vascular Grafts Based on Elastin-Like Recombinamers for *in situ* Tissue Engineering

**DOI:** 10.3389/fbioe.2019.00340

**Published:** 2019-11-19

**Authors:** Alicia Fernández-Colino, Frederic Wolf, Stephan Rütten, Thomas Schmitz-Rode, Jose Carlos Rodríguez-Cabello, Stefan Jockenhoevel, Petra Mela

**Affiliations:** ^1^Department of Biohybrid & Medical Textiles (BioTex), AME-Institute of Applied Medical Engineering, Helmholtz Institute, RWTH Aachen University, Aachen, Germany; ^2^Electron Microscopy Facility, Uniklinik RWTH Aachen, Aachen, Germany; ^3^AME-Institute of Applied Medical Engineering, Helmholtz Institute, RWTH Aachen University, Aachen, Germany; ^4^Bioforge Lab, University of Valladolid, CIBER-BBN, Valladolid, Spain; ^5^AMIBM-Aachen-Maastricht-Institute for Biobased Materials, Maastricht University, Geleen, Netherlands; ^6^Medical Materials and Implants, Department of Mechanical Engineering and Munich School of BioEngineering, Technical University of Munich, Garching, Germany

**Keywords:** biohybrid scaffolds, elastin-like recombinamers, textile technical components, vascular grafts, off-the-shelf implants

## Abstract

Vascular disease is a leading cause of death worldwide, but surgical options are restricted by the limited availability of autologous vessels, and the suboptimal performance of prosthetic vascular grafts. This is especially evident for coronary artery by-pass grafts, whose small caliber is associated with a high occlusion propensity. Despite the potential of tissue-engineered grafts, compliance mismatch, dilatation, thrombus formation, and the lack of functional elastin are still major limitations leading to graft failure. This calls for advanced materials and fabrication schemes to achieve improved control on the grafts' properties and performance. Here, bioinspired materials and technical textile components are combined to create biohybrid cell-free implants for endogenous tissue regeneration. Clickable elastin-like recombinamers are processed to form an open macroporous 3D architecture to favor cell ingrowth, while being endowed with the non-thrombogenicity and the elastic behavior of the native elastin. The textile components (i.e., warp-knitted and electrospun meshes) are designed to confer suture retention, long-term structural stability, burst strength, and compliance. Notably, by controlling the electrospun layer's thickness, the compliance can be modulated over a wide range of values encompassing those of native vessels. The grafts support cell ingrowth, extracellular matrix deposition and endothelium development *in vitro*. Overall, the fabrication strategy results in promising off-the-shelf hemocompatible vascular implants for *in situ* tissue engineering by addressing the known limitations of bioartificial vessel substitutes.

## Introduction

Cardiovascular disease is the most common cause of death worldwide (Townsend et al., 2016). The progressive constriction and stiffening of the blood vessels requires, in complex conditions, bypass surgery (Townsend et al., 2016). Native autologous vein and artery segments remain the gold standard as grafts for this procedure, but they are not always available due to a previous harvest, anatomical variability or disease progression. Furthermore, the failure rate of saphenous vein grafts, the most widely used conduits for coronary artery bypass, reaches 25% during the first 12–18 months after surgery, clearly showing the need for better solutions (Hess et al., 2014). The synthetic vascular prostheses available today are made of expanded polytetrafluoroethylene (GORE-TEX^®^), poly(ethylene terephthalate) (Dacron) or polyurethane. These vascular prostheses lack the remodeling and growing capabilities of living tissues and are not suitable as small-diameter substitutes (<6 mm) because of poor blood compatibility and compliance mismatch that lead to thrombosis and intimal hyperplasia (Salacinski et al., 2001; Zilla et al., 2007).

Tissue engineering (TE) approaches have the potential to provide alternative solutions to current underachieving artificial replacements. Vascular TE strategies are increasingly moving from a classical to an *in situ* approach also known as directed endogenous regeneration. Here, cell-free scaffolds are implanted to be colonized and remodeled endogenously, resulting in autologous vessel substitutes (Wissing et al., 2017). Off-the-shelf availability, lower regulatory burden for clinical translation and no need for tissue harvest for cell isolation are major advantages of *in situ* TE with respect to the classical cell-based approach. On the other hand, this strategy places strong demands on the implant's material and fabrication method as the graft has to perform adequately upon implantation, which means it has to be able to withstand the systemic circulation, be hemocompatible, and provide a microenvironment suitable for cell infiltration and tissue generation (Billiet et al., 2012).

Electrospinning is a widely employed technique to obtain biodegradable grafts (Park et al., 2019), which has been in some cases combined with surface functionalization to promote endothelialization and improve hemocompatibility (Zhao et al., 2019). However, electrospun scaffolds typically suffer from poor cellular infiltration because of the dense fibrous network (Zhong et al., 2012). Salt leaching (Lee et al., 2011) and freeze drying (Sugiura et al., 2016) techniques have been used to create interconnected porous architectures which are advantageous in term of cellular colonization and matrix deposition. However, the initial poor mechanical properties of the porous scaffold might compromise a safe implantation (Lee et al., 2011). Despite encouraging results demonstrating the potential of *in situ* TE (Wissing et al., 2017), control over the properties of the developed grafts remains at best partial.

The development of vascular grafts that combine both the elasticity to allow an energy-efficient transmission of the pulsatile blood flow with the strength to withstand the blood pressure is particularly challenging, as burst strength and compliance are often inversely related (Sarkar et al., 2007). While compliance mismatch can lead to intimal hyperplasia, low patency and consequent graft failure (Abbott et al., 1987; Trubel et al., 1995; Ballyk et al., 1998; Salacinski et al., 2001; Kannan et al., 2005; Sarkar et al., 2006), compliance as endpoint in vascular graft design has been many times overlooked in the literature (Pashneh-Tala et al., 2015). Additionally, because of the variability of this property between different vessels, vascular TE should ideally aim at developing compliant vessel substitutes with mechanical properties tailored to the intended implantation site. The ability of cardiovascular structures to deform elastically with the pressure pulse is crucial to maintain their function over time and relies on the high percentage of elastin in the native vessel wall, accounting for 50% of the dry weight of major arteries (Rosenbloom et al., 1993). The lack of functional elastin synthesis by the cells is a major drawback of tissue-engineered vascular grafts (TEVGs), which results in aneurysm formation as a major failure mechanism (Wissing et al., 2017). This makes elastin and elastomeric materials highly attractive for the engineering of vessel substitutes.

Our approach is to develop a platform that enables an *ad-hoc* vascular fabrication according to the specific target by combining the elastic properties provided by the elastin-like recombinamers (ELRs) with the mechanical support of technical textile components. The ELRs are a family of artificial polymers bioinspired by the pentapeptide VPGVG present in the natural elastin (MacEwan and Chilkoti, 2010; Girotti et al., 2011). They are positioned midway between natural products and synthetic polymers, as they are engineered in a controlled and highly reproducible way while still maintaining inherent properties of the natural elastin such as elastic mechanical behavior, hemocompatibility, and bioactivity (Arias et al., 2014). The textile components consist of a warp knitted polyvinylidene fluoride (PVDF) mesh to confer suturability, long term stability and, in combination with an electrospun polycaprolactone (PCL) mesh, burst strength and tunable compliance.

Evaluation of the obtained biohybrid vascular grafts included burst strength, compliance, suture retention, cell infiltration, hemocompatibility, extracellular matrix (ECM) deposition, and endothelialization.

## Materials and Methods

### Complement Convertase

Complement activation was measured using the Complement Convertase Kit (CCA, HaemoScan, Groningen, Netherlands), which follows the international standard ISO 10993/Part 4 to evaluate the biocompatibility of biomaterials and medical devices. The assay was performed according to the manufacturer's protocol. Briefly, 1 cm^2^ samples of different materials (i.e., the click-ELR test material, and MS (medical steel) and PDMS (polydimethylsiloxane) reference materials provided with the kit as internal controls) were incubated first with plasma for 15 min, and then with a chromogenic C5 convertase substrate for 24 h. Cleavage of the substrate by C5 convertases present on the surface of the tested materials was quantified by measuring the optical density at 405 nm in a microplate reader (Infinite M200, Tecan).

### Thrombogenicity

Blood (12 mL) was drawn from healthy volunteers and mixed with 3.2% sodium citrate using an S-Monovette CPDA1 device (Sarstedt, Germany) at a volumetric ratio of 1:9. Six mL of blood were centrifuged at 270 × *g* for 15 min while the other 6 mL were centrifuged at 2,000 × *g* for 15 min. The resulting supernatants were mixed at a volumetric ratio of 1:1 to obtain platelet-rich plasma (PRP). Each sample was placed in a 96-well plate, and 100 μL of PRP was added and incubated for 1 h at 37°C. The samples were then gently washed with PBS and fixed in 3% glutaraldehyde in 0.1 M Sorenson's buffer (pH 7.4) for SEM. Evaluation of platelet adhesion was carried out based on the SEM images by assessing images from three independent experiments, and for each experiment, three fields of 350 μm^2^ each were analyzed.

### Scaffold Fabrication

We used two ELRs, namely VKVx24 and HRGD6. VKVx24 is a structural recombinamer with no bioactive sequence, whereas HRGD6 is a recombinamer containing a RGD adhesion sequence (Table 1). The lysine residues of each ELR were chemically modified as previously reported (Gonzalez de Torre et al., 2014) to introduce cyclo-octyne and azide groups, resulting in VKVx24-cyclo-octyne and HRGD6-azide, respectively.

**Table 1:** The amino acid sequences of each recombinamer and the corresponding reactive groups used for click chemistry.

**ELR**	**Amino acid sequence**	**Reactive group**
VKVx24-c	MESLLPVGVPGVG[VPGKG(VPGVG)_5_]_23_ VPGKGVPGVGVPGVGVPGVGVPGV	Cyclo-octyne
HRGD6-a	MGSSHHHHHHSSGLVPRGSHMESLLP[(VPGIG)_2_(VPGKG)(VPGIG)_2_]_2_AVTGRGDSPASS [(VPGIG)_2_(VPGKG)(VPGIG)_2_]_2_	Azide

*The reactive groups were incorporated by chemical modification of the lysine residues. VKVx24-c refers to VKVx24-cyclo-octyne and HRGD6-a to HRGD6-azide*.

For the fabrication of the macroporous ELR-based scaffolds, we used the salt leaching/gas foaming technique that we recently developed (Fernandez-Colino et al., 2018). Briefly, each ELR component was dissolved to a concentration of 75 mg/mL in a 1:1 (v/v) mixture of PBS and ethanol. NaHCO_3_ particles (diameter <100 μm) were added to each ELR solution at a recombinamer/salt weight ratio of 1:10. Each preparation was loaded into a syringe, and injected into the mold using a dual chamber syringe applicator. The mold consisted of a gas-permeable outer silicone tube (inner diameter = 6.4 mm; Ismatec) and an inner metal cylinder (outer diameter = 3 mm) placed coaxially to the silicone tube. A PVDF mesh was positioned in the annular space between the cylinders. The mesh was produced at the Institute of Textile Engineering (Institut für Textiltechnik) of the RWTH Aachen University (Aachen, Germany). The space between cylinders was then filled with the ELR components. After incubating for 30 min at 37°C, the mold was removed, and the click-ELR scaffold was released and placed in 3 M citric acid for 30 min in an ultrasound bath (Martin et al., 2009). The scaffolds were washed with aqueous phosphate-buffered saline (PBS) to eliminate residual ethanol, salts and acid, and then they were enclosed in an outer PCL sheath by directly electrospinning on the graft. The PCL (GMP grade, Purasorb PC12, Corbion Purac Gorinchem, Netherlands) was dissolved at a concentration of 14% in a 1:3 (v/v) mixture of methanol and chloroform, which was infused through a 27-gauge needle at a constant flow rate of 3 mL/h. A voltage difference of 24 kV was applied between the needle and the collector, which were placed 18 cm apart. The temperature and the humidity were set at 32°C and 20%, respectively. Finally, the vascular grafts were sterilized by washing with 70% ethanol for 30 min and stored in fresh 70% ethanol overnight. The scaffolds were then incubated in PBS for 2 × 30 min and 1× 16 h.

### Scanning Electron Microscopy (SEM)

Samples were fixed in 3% glutaraldehyde in 0.1 M Sorenson's buffer (pH 7.4) at room temperature for 1 h. They were rinsed with 0.2 M sodium phosphate buffer (pH 7.39, Merk) and dehydrated in 30, 50, 70, and 90% acetone, and then three times in 100% acetone, for 10 min each. After critical point drying in CO_2_, the samples were sputter-coated (Leica EM SC D500) with a 20 nm gold–palladium layer. Images were captured using an ESEM XL 30 FEG microscope (FEI, Philips, Eindhoven, Netherlands) with an acceleration voltage of 10 kV. The pore size was determined using ImageJ software by analyzing three different regions, with 50 measurements taken in each region. Some of the SEM images were colored with MountainsMap SEM software courtesy of Digital Surf, France.

### Burst Strength

Burst strength values were measured in a custom-made burst strength chamber equipped with a pressure sensor (Jumo Midas pressure transmitter; JUMO GmbH & Co. KG) and a peristaltic pump (IPC Ismatec, IDEX Health & Science GmbH). Samples (*n* = 3) with areas of 1 cm^2^ were placed in the burst chamber and exposed to increasing pressure by pumping PBS at a constant rate of 5 mL/min until the structural failure of the sample was detected as a sudden drop in the pressure recorded by LabVIEW (National Instruments). The highest pressure measured before failure was defined as the burst strength value.

### Suture Retention

Suture retention strength was measured by inserting a 7–0 prolene suture 1 mm from the edge of each graft. The suture was fixed on the upper hook of a Z2.5 Zwick/Roell tensile tester (Zwick GmbH & CO. KG; Ulm; Germany) and the grafts were immobilized on the lower hook. The system was then strained at a speed of 50 mm/min (in accordance with ISO guidelines) to pull the suture away from the graft, and the required force was recorded. Three samples were measured for Elastingraft_eM, and six for the ElastinGraft_0.

### Compliance and Stiffness Index

The vascular grafts were placed in a bioreactor system that comprised a custom-made chamber made of polyoxymethylene (POM; Licharz GmbH, Buchholz, Germany) with transparent poly(methyl methacrylate) (PMMA) sides and an optical micrometer [model LS-7030(M), Keyence Deutschland GmbH, Neu-Isenburg, Germany] to measure the diameter of the vascular graft by emitting a parallel beam of green light to the graft and assessing its shadow. Additionally, the system featured silicone tubes (Ismatec), a flow sensor (SonoTT™ Flowcomputer, em-tec GmbH), an adjustable resistance to control the flow within the system, a pressure sensor (Xtrans, Codan pvb Medical GmbH), and a small centrifugal pump (702-6882, RS components), which was controlled by a custom-developed control unit (Wolf et al., 2018).

The flow-loop was filled with phosphate buffer solution (Gibco, pH: 7.4). The mean pressure was created by a continuous flow generated by the centrifugal pump, in combination with an adjustable resistance. The pressure pulses (20/60, 30/70, 40/80, 50/90, 60/100, 70/110, 80/120, 90/130, and 110/150 mmHg) were created by modulation of the pulse-free flow, due to changing the constant input voltage, using a sinusoidal function. The values of pressures and corresponding graft diameters were recorded with a custom-developed LabVIEW program (LabVIEW 7.1; National Instruments). The average values of the compliance were estimated from such values, using the following equation

$$\begin{array}{l} \text{C}=\left[\left(\left({\text{D}}_{2}-{\text{D}}_{1}\right)/{\text{D}}_{1}\right)\times 10^{4}\right]/\left({\text{P}}_{2}-{\text{P}}_{1}\right) \end{array}$$

where P_1_ is the lowest internal pressure, P_2_ is the highest internal pressure, D_1_ is the diameter at the pressure P_1_ and the D_2_ is the diameter at the pressure P_2_. Pressure–diameter measurements were also used to calculate the dimensionless stiffness index (Soletti et al., 2010):

$$\begin{array}{l} \beta =\text{ln}\left({\text{P}}_{2}/{\text{P}}_{1}\right)/\left[\left({\text{D}}_{2}-{\text{D}}_{1}\right)/{\text{D}}_{1}\right] \end{array}$$

### Ultrasound Monitoring

The stretching and recoiling capability of the vascular grafts was also visualized with an ultrasound system, Vivid-I (S/N 3642VI General Electric), with a linear probe (8L-RS, General Electric).

### Cell Isolation and Culture

Smooth muscle cells (SMCs) were isolated from the veins of human umbilical cords as previously described (Moreira et al., 2015). Briefly, the vein was washed with PBS (Gibco) before endothelial cells were removed using 1 mg/mL collagenase (Sigma) and the adventitia was stripped off. To isolate SMCs, the digested vein was minced into 1-mm rings and then bathed in Dulbecco's modified Eagle's medium (DMEM) supplemented with 10% FBS and 1% antibiotic/antimycotic solution (all reagents from Gibco). To obtain a sufficient cell number, the cells were serially passaged using 0.05% trypsin/0.02% EDTA (Gibco) and cultured at 95% humidity and 37°C in a 5% CO_2_ atmosphere. SMC phenotype was verified by the presence of alpha-SMA positive cells and the absence of von Willebrand factor expression by immunocytochemistry. Cell passages 4–7 were used for all experiments. To study the interaction between the SMCs and the click-ELR vascular grafts, the ElastinGrafts were coaxially introduced in a silicone tube clamped at one end. One mL of the cell-suspension at 3 × 10^6^ cells/mL was then introduced into the silicone tube and the other end was also clamped. The system was attached to a mandrel connected to the head of a roller pump (MCP Process; Ismatec), and rotated along the longitudinal axis for 6 h following a cycle of 20 s in rotation at 1 rpm and 200 s in static to allow cell attachment. The remaining cell suspension was then removed, and the graft was placed in fresh culture medium. Cell behavior and ECM deposition were investigated after 12 days of static culture by SEM and immunohistochemistry.

Endothelial progenitor cells (EPCs) were isolated from human adult peripheral blood mononuclear cells. Anticoagulated blood of healthy donors was carefully added to the separating solution Histo-Paque-1077 (Sigma-Aldrich, St. Louis, Missouri), and centrifuged at 400 × *g* at room temperature for 30 min. The layer containing mononuclear cells was washed twice by gently mixing with PBS, and subsequent centrifuging at 250 × *g* for 10 min. The cell pellet was resuspended with 15 mL of endothelial cell growth medium (Endothelial Cell Growth Medium MV2; PromoCell, Heidelberg, Germany), containing epidermal growth factor (5 ng/mL), basic fibroblast growth factor (10 ng/mL), insulin-like growth factor (20 ng/mL), vascular endothelial growth factor 165 (0.5 ng/mL), ascorbic acid (1 μg/mL), and hydrocoecisone (0.2 μg/mL), and transferred into T-75 culture flask pre-coated with human fibronectin (1 mg/cm^2^, Sigma-Aldrich, St. Louis, MO). The cells were incubated at 37°C in a humidified atmosphere containing 5% CO_2_. When EPCs colonies were formed, they were trypsinized [0.05% trypsin/0.02% ethylenediaminetetraacetic acid solution (Gibco, Karlsruhe, Germany)] and transferred to T-25 culture flask pre-coated with type I rat-tail collagen (5 mg/cm^2^, BD Biosciences, San Jose, CA). When cells reached 70–80% of confluence, they were trypsinized and transferred into T-75 culture flasks. The phenotype of EPCs was corroborated by immunocytochemical staining for the markers vWF and CD31 and by flow cytometric analysis, which showed positive signals for the EC markers CD31 and CD144 and negative signals for the monocytic markers CD14 and CD45. Cells up to eight passages were used for all experiments.

To study the ability of the click-ELR grafts to support endothelialization, the scaffolds were incubated with a suspension of EPCs (3 × 10^6^ cells/mL) while rotating at 1 rpm for 6 h as described for SMC seeding. Then, the scaffolds were cultured in static for 1 and 4 days and subsequent investigated by SEM and immunohistochemistry followed by confocal microscopy.

### Immunohistochemistry

Native human umbilical cord and click-ELR scaffolds seeded with SMCs were fixed in Carnoy's solution and embedded in paraffin before sectioning. The sections were deparaffinized, non-specific sites were blocked and the cells permeabilized by incubating in 5% normal goat serum (NGS, Dako) in 0.1% Triton-PBS. The sections were incubated for 1 h at 37°C with the primary rabbit anti-collagen I antibody (R 1038, Acris) diluted 1:200. The sections were then incubated for 1 h at 37°C with a fluorescein-conjugated secondary antibody produced in rabbit (A11008, Molecular Probes) diluted 1:400. The native human umbilical cord served as a positive control. For negative controls, samples were incubated in diluent and the secondary antibody only. Tissue sections were counterstained with 4′,6-diamidino-2-phenylindole (DAPI) nucleic acid stain (Molecular Probes). Samples were observed under a microscope equipped for epi-illumination (AxioObserver Z1, Carl Zeiss GmbH). Images were acquired using a digital camera (AxioCam MRm, Carl Zeiss GmbH). Click-ELR scaffolds seeded with EPCs were fixed with methanol-free formaldehyde (Roth, Karlsruhe, Germany) at 4% for 1 h at room temperature. Non-specific sites were blocked and the cells permeabilized by incubating in 5% normal goat serum (NGS, Dako) in 0.1% Triton-PBS. The samples were incubated overnight at 4°C with the primary mouse anti-CD31 antibody (P8590, Sigma) diluted 1:100. The samples were then incubated for 1 h at room temperature with AlexaFluor 594 anti-mouse antibody produced in goat (A11005, Invitrogen) diluted 1:400. Images were acquired using a Zeiss LSM 710 confocal laser scanning microscope.

### Statistical Analysis

Differences between three or more groups were assessed using one-way ANOVA with Tukey's *post-hoc* test. Statistical difference between two groups was evaluated by the Student's *t*-test. A significance level *p* = 0.05 was set for all the tests. In the figures, statistical significance is denoted as ^*^ for *p*-value ≤ 0.05, ^**^ for *p*-value ≤ 0.01, ^***^ for *p*-value ≤ 0.001.

## Results and Discussion

### *In vitro* Hemocompatibility Testing

**Figure 1:**
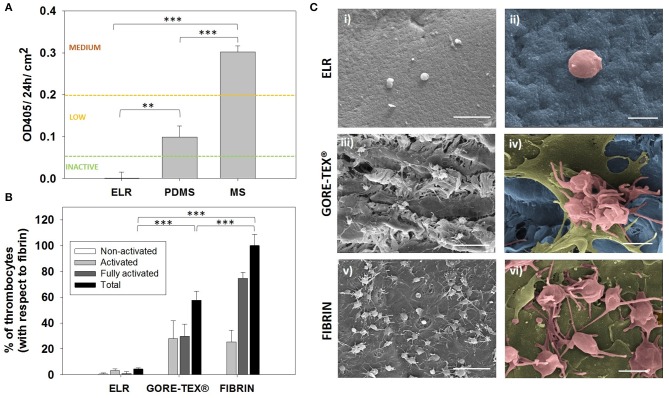
Hemocompatibility assessment. **(A)** Hemocompatibility of ELR-gels assessed using the C5 complement convertase activity. Control materials used for comparison: medical steel (MS) and polydimethylsiloxane (PDMS). **(B)** Evaluation of adhered platelets based on SEM images. Results are plotted as the mean of three different experiments ± standard deviation. **(C)** SEM images of (i,ii) ELR hydrogel, (iii,iv) GORE-TEX^®^, and (v,vi) fibrin-gel after 1 h in contact with platelet rich plasma. Activated and fully activated platelets were covering fibrin and GORE-TEX^®^ surfaces, while ELR surfaces presented minimal platelet adhesion. In the images ii, iv and vi, the ELR and GORE-TEX^®^ scaffolds were colored in blue, the platelets with a spread morphology in gold and the more rounded platelets in salmon. Scale bars: (i,iii,v): 10 μm; (ii,iv,vi) 2 μm.

The concept of ready-to-use vascular grafts implies the absence of any *in vitro* seeding of the device prior implantation. Such device will be in direct contact with blood before a native endothelium covers its surface, and consequently, the first requirement that the base material has to meet is to be hemocompatible. Therefore, we first evaluated the hemocompatibility of the ELRs by assessing the activation of the complement system. For this purpose, we used a complement convertase assay that complies with the international standard ISO 10993/Part 4. Results and given thresholds to evaluate the activation are indicated in Figure 1A. The ELR hydrogels behaved as a complement non-activating biomaterial (<0.06 OD/24 h/cm^2^), giving the lowest value among all the tested materials (i.e., MS and PDMS).

The reaction of blood components to the presence of ELR was further studied by performing a thrombogenicity test. A high number of activated and adherent platelets were present on the GORE-TEX^®^ and fibrin control after 1 h of PRP contact, whereas minimal platelet adhesion was detected on the surface of the ELR-samples (Figure 1B). Moreover, the platelets on the GORE-TEX^®^ and fibrin samples presented either extended pseudopodia (colored in salmon Figure 1Civ,vi) or “fried egg” morphology (colored in gold in Figure 1Civ,vi), indicative of activated and highly activated states, respectively (Figure 1C), whereas the few platelets on the ELR samples presented a rounded morphology, with few pseudopodia (correlated with a lower activation state) (Goodman et al., 1989; Sorrentino et al., 2016). These results agree with previous studies which propose elastin and elastin-like polymers as excellent candidates for the fabrication of blood-contacting materials (Ito et al., 1998; Woodhouse et al., 2004; Jordan et al., 2007; de Torre et al., 2015) and support the use of ELRs for the fabrication of vascular grafts as cell-free implants which will be in direct contact with the blood before endothelialization.

### Fabrication of the Click-ELR Vascular Grafts (ElastinGrafts)

**Figure 2:**
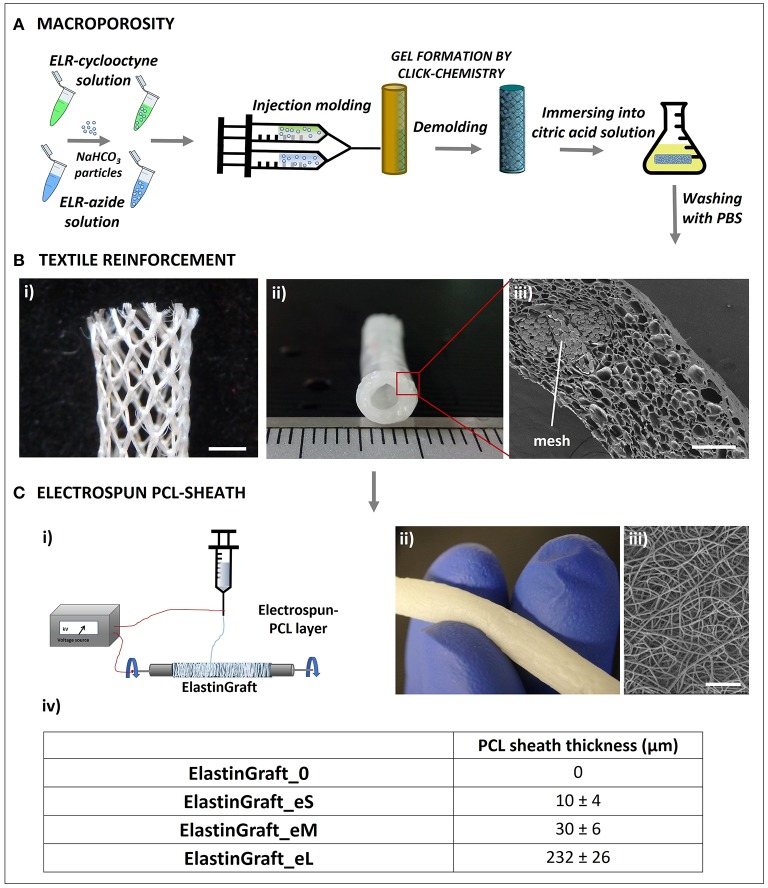
Biofabrication scheme of the ElastinGrafts: textile-reinforced macroporous vascular grafts based on ELRs. **(A)** Procedure used to obtain the macroporous click-ELR vascular graft. **(B)** Images of the textile-reinforced macroporous vascular graft: (i) PVDF-textile reinforcement to provide mechanical strength. (ii) Elastin graft. (iii) Cross-sectional SEM image of the macroporous ELR-vascular graft. **(C)** Electrospun PCL-sheath. (i) Sketch of the electrospinning procedure. (ii) Macroscopic image of the ElastinGraft enclosed with a PCL-sheath. (iii) SEM images of the electrospun sheath around the ELR-scaffold. (iv) Different versions of the ElastinGrafts differing in the thickness of the electrospun PCL-sheath. Specifically, “0” refers to “no e-spun layer”; “eS” to “the smallest thickness of the e-spun layer,” “eM” to “the intermediate thickness of the e-spun layer” and “eL” to “the largest thickness of the e-layer.” Scale bars: **(B)** (i) 2 mm; (iii) 200 μm; **(C)** (iii) 50 μm.

Vessel substitutes were fabricated as schematically shown in Figure 2. Microscopic inspection of the ElastinGrafts revealed that (i) a highly porous structure was obtained throughout the whole construct, with a mean pore size of 33 μm, which has been indicated as adequate for the interaction with smooth muscle cells (SMCs) (Lee et al., 2011); (ii) the textile was well embedded within the ELR (Figure 2Biii) without any gaps in between the mesh pores (Supplementary Figure 1); and (iii) a fibrillar sheath enclosed the vascular graft (Figure 2C). The scaffold structure determines both the mechanical performance and the cell behavior of the implant (e.g., cell ingrowth and ECM formation), and ultimately its overall success. The porous microstructure should provide a template for cell ingrowth, whereas the textile reinforcement and the electrospun sheath should provide adequate mechanical properties. These aspects were addressed in the next set of experiments.

### Mechanical Characterization

A vessel substitute *in vivo* must withstand the pulsatile blood pressure and flow, and must display enough strength to provide a safe implantation, while maintaining the capability of recoiling after stretching. To confirm whether our fabrication approach achieved an implantable device, we carried out burst strength, compliance and suture retention tests.

**Figure 3:**
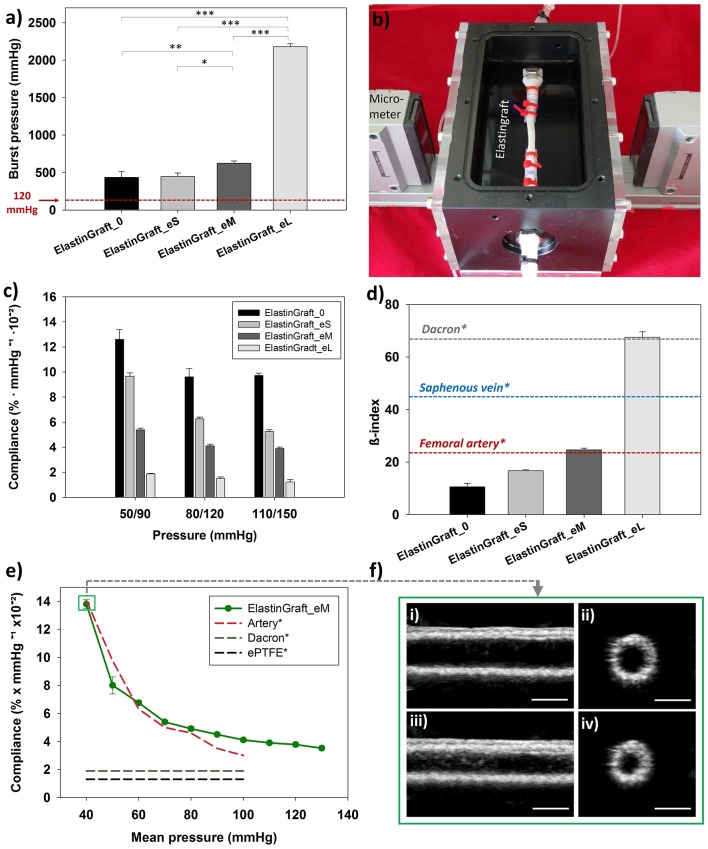
Mechanical characterization of the ElastinGrafts. **(a)** Burst strength. **(b)** Vascular graft chamber used for assessing the compliance. **(c)** Compliances of the different ElastinGrafts at low (50–90 mmHg), normal (80–120 mmHg), and high pressure (110–150 mmHg). **(d)** Comparison of stiffness (β-index) between the ElastinGrafts, native blood vessels [value of the human femoral artery taken from Emoto et al. (1998) and value of the human saphenous vein taken from Zhang et al. (2017)] and Dacron [value taken from Tai et al. (2000)]. Values taken from literature are marked with an asterisk (^*^). **(e)** Compliance-mean pressure curve for the ElastinGraft_eM, artery, Dacron, and ePTFE. The values from the human external iliac artery, Dacron and ePTFE were adapted from Tai et al. (2000), Sarkar et al. (2006). **(f)** Ultrasound images of the ElastinGraft_eM in the simulated systolic low pressure of 60 mmHg (i,ii) and diastolic low pressure of 20 mmHg (iii,iv). The complete videos for the low (20/60 and 50/90 mmHg), normal (80/120 mmHg), and higher pressure ranges (110/150 mmHg) are available as supporting information (Supplementary Video S1).

The burst strength of the vascular grafts was proportional to the PCL-sheath thickness, as shown in Figure 3a, and ranged from 436 ± 76 mmHg (ElastinGraft_0, Figure 2vii) to 2181 ± 37 mmHg (ElastinGraft_eL, Figure 2vii), which approximates the value of human saphenous vein (2250 mmHg) (Sarkar et al., 2006). Notably, all grafts displayed adequate strength (4–12 times the physiological pressure of 120 mmHg) for implantation. Historically, vascular engineering has aimed at developing vessel conduits that overmatch the values of native vessels (L'Heureux et al., 2006; Zhang et al., 2006; Drilling et al., 2009; McAllister et al., 2009). These supraphysiological burst strengths are normally the result of a long bioreactor conditioning phase producing dense tissues mainly composed of collagen and lacking elastin. However, there is an increasing number of studies showing that vascular grafts with a less mature matrix and featuring burst strength values lower than those displayed by target native vessels are still adequate for a safe implantation *in vivo* (Koch et al., 2010; Lee et al., 2011; Fukunishi et al., 2016; von Bornstadt et al., 2018) while favoring a more extensive *in situ* remodeling. The necessity of evaluating the burst pressure is indicated in the ISO guidelines 7198, but no target burst pressure values are given, and the question on which threshold pressure is required for a safe implantation remains open.

Matching compliance is of key importance to avoid hemodynamic flow changes across anastomosis, which have been associated with intimal hyperplasia, low patency, and consequent graft failure (Abbott et al., 1987; Trubel et al., 1995; Ballyk et al., 1998; Salacinski et al., 2001; Sarkar et al., 2006). Besides these effects at the graft site, the compliance miss-match can also lead to deleterious effects on the cardiac function. For example, the ascending aorta replacement with non-compliant grafts can result in aortic valve insufficiency and left ventricular hypertrophy (Spadaccio et al., 2016). The dynamic compliances of the vascular grafts at different pressure ranges are summarized in Figure 3. All the ElastinGrafts, except the ElastinGraft-eL, displayed higher compliances values than the commercially available grafts made of Dacron (1.9 ± 0.3% mmHg^−1^ × 10^−2^) and ePTFE (1.6 ± 0.2% mmHg^−1^ × 10^−2^) (Salacinski et al., 2001), which evidences the potential for energy-efficient transmission of the pulsatile flow provided by their elastic structure. Notably, the ElastinGraft_eM (4.11% mmHg^−1^ × 10^−2^) compares well with the compliance of human arteries [4.5–6.2% mmHg^−1^ × 10^−2^ (L'Heureux et al., 2006)] in the 80–120 mmHg range.

Importantly, the arterial tissue is anisotropic, that is, it does not obey Hook's law in response to cyclical blood pressure changes. As such, the relationship between the intraluminal pressure and the degree of radial deformation is not linear. The increased compliance found at lower physiological pressures is of key importance to preserve the pulsatile energy in situations of hemorrhagic shock (systolic blood pressure <90 mmHg) (Salacinski et al., 2001). However, this anisotropy is not displayed by prosthetic grafts, which typically present a constant compliance regardless the pressure, and they may, therefore, compromise patient-safety in case of a response of shock. The ElastinGrafts were able to recapitulate this anisotropic behavior, presumably because of the elastin-like structure, and specifically the ElastinGraft_eM mirrored the values of the native artery in the whole range of mean pressures tested (Figure 3e and Supplementary Video 1). The stiffness of the vascular grafts was also analyzed by calculating the β-index, which is a parameter less dependent on blood pressure (Mackenzie et al., 2002) and, therefore, facilitates the comparison among the performance of different grafts reported in the literature. The β-index of all but the ElastinGraft-eL was much lower than that of the human saphenous vein (45 ± 12.5) (Zhang et al., 2017) and Dacron (67.8 ± 32.3) (Tai et al., 2000), and importantly, the ElastinGraft_eM's β-index (24.6 ± 0.64) matched that of the femoral artery (23.7 ± 0.8) (Emoto et al., 1998).

The compliance of the ElastinGrafts can be tuned over a wide range of values encompassing those of native vessels, as shown in Figure 3. The mechanical properties of the implant can, therefore, be tailored in a vessel specific manner. Considering that later steps in the vascular grafts' evaluation will inevitably involve animal trials, the possibility of tuning the compliance accordingly to that of the target animal vessel may result in a more accurate evaluation of its performance. For example, the native carotid artery of the sheep, which is a broadly used implantation model for vessel evaluation, has a compliance of approximately 12–14% (Fukunishi et al., 2016), which compares better with our ElastinGraft_0 (9.6% mmHg^−1^ × 10^−2^) than with the ElastinGraft_eM (4.11% mmHg^−1^ × 10^−2^). Whether the initial compliance should match that of the target vessel is, however, an open question that only further *in vivo* testing can elucidate as the *in situ* remodeling of the graft after implantation may result in changes in the mechanical properties. Collagen deposition, for example, may lead to an increase in the stiffness of the graft and to a concomitant decrease in the compliance (Uchida et al., 1989; Zilla et al., 2007; Fukunishi et al., 2016). Under such scenario, the use of implants with initial compliance values higher than those of the target vessels may be recommended. However, how the scaffold degradation can additionally influence the compliance by, for example, counterbalancing the increased collagen amount, belongs as well to the gap of knowledge that represents a challenging aspect of the design of any implant based on the concept of endogenous tissue growth (Pashneh-Tala et al., 2015; Wissing et al., 2017). Nevertheless, the platform that we developed here enables to meet a desired compliance once defined as design endpoint (Ozolanta et al., 1998).

The suture retention force of the ElastinGraft_0 and the ElastinGraft_eM was 2.1 ± 1.4 and 2.8 ± 1.8 N, respectively. These values compare well with the human internal mammary arteries (1.4 N) and human saphenous veins (1.8 N) (Konig et al., 2009). The absence of statistical difference between both ElastinGrafts (*p* > 0.05) suggests that the PCL layer does not play a significant role in providing suture retention which is, therefore, attributable to the knitted mesh. The high standard deviation reflects the structure of the knit pattern of the PVDF mesh (Figure 2B). Overall, these results indicate that the newly developed grafts have a suture retention strength adequate for direct anastomosis (Wu et al., 2012). PVDF is one of the most popular polymers in textile biomedical engineering due to its processability, mechanical properties, biostability, and minimal tissue response (Klinge et al., 2002). The mechanical reinforcement provided by a knitted textile has been previously exploited in classically tissue-engineered cell-based vascular grafts implanted as arteriovenous shunts (Mertens et al., 2015) and as interpositional grafts in the systemic circulation of a large animal model (Koch et al., 2010; Wolf et al., 2019).

### Cell Ingrowth and Extracellular Matrix Production

**Figure 4:**
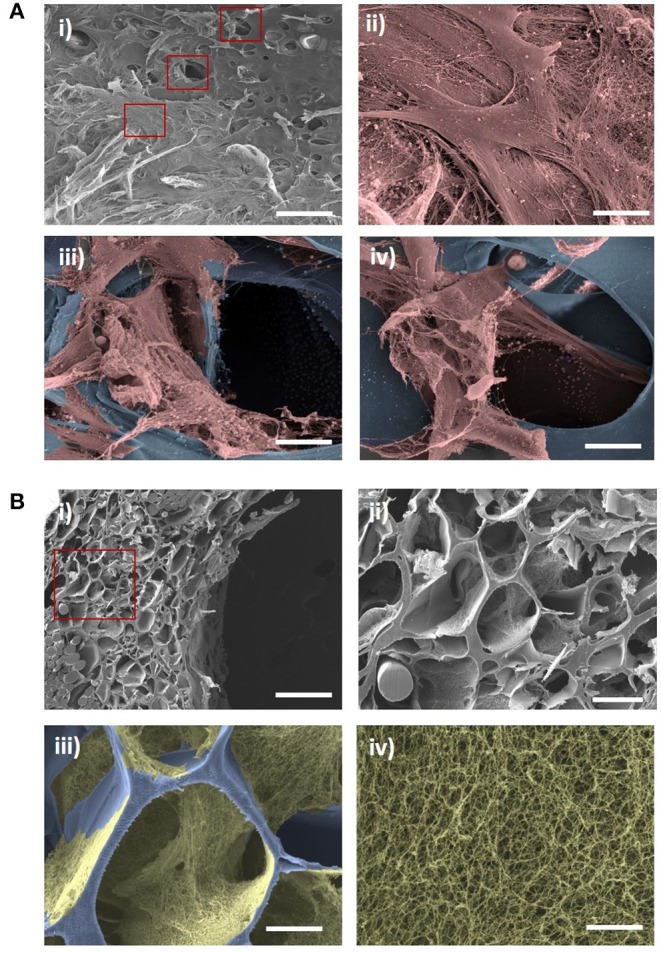
SEM evaluation of cell ingrowth and ECM production after *in vitro* culture of the ElastinGrafts with SMCs. **(A)** Luminal surface of the ElastinGraft; (i) overview and (ii) detailed view of the SMCs covering the luminal surface of the graft. (iii,iv) Detailed views of SMCs infiltrating the scaffold. In images **(A)** (ii–iv) the ELR-scaffold was colored in blue and the SMCs in salmon. **(B)** (i,ii) Porous structure of ElastinGraft visualized at a cross-section. (iii) Representative pore covered with deposited ECM. (iv) Zoomed-in view of the fibrillar ECM. In the images **(B)** (iii,iv) the ELR-scaffold was colored in blue and the fibrillar ECM was colored in gold. Scale bars. **(A)** (i) 200 μm: **(B)** (ii–iv) 20 μm. **(B)** (i) 200 μm; (ii) 50 μm; (iii) 20 μm; (iv) 2 μm.

**Figure 5:**
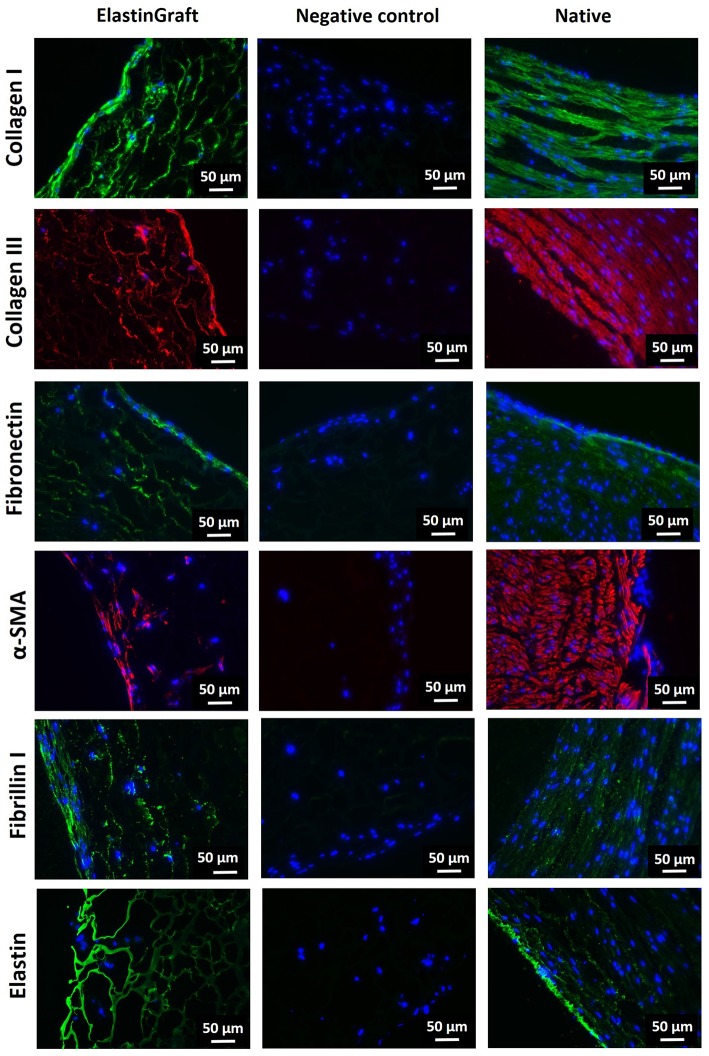
Immunohistochemical evaluation of cell ingrowth and ECM production after *in vitro* culture of the ElastinGrafts with SMCs. Analysis of the ElastinGraft and a native human artery from the umbilical cord, showing staining for alpha-SMA, collagen I, collagen III, fibronectin, fibrillin I, and elastin. Negative controls (absence of the primary antibody) for all markers showed undetectable levels of staining.

To determine whether the ElastinGrafts can support cell colonization and ECM deposition, we cultured them *in vitro* with SMCs. SEM and immunohistochemistry confirmed cell infiltration into the walls of the vascular grafts, and ECM deposition was shown after 12 days of static culture *in vitro* (Figures 4, 5). Specifically, the SEM images revealed the presence of fibrillar structures attributable to the secreted ECM in most of the pores along the cross-section of the vascular graft wall (Figure 4B).

Immunohistochemistry revealed α-SMA positive-stained cells, which corroborated their SMC phenotype, besides the deposition of collagen I, collagen III, fibronectin and fibrillin I (Figure 5). Fibrillin I is an elastic matrix-forming protein, whose expression precedes that of elastin (Votteler et al., 2013) and guides elastogenesis (Mariko et al., 2011). There was also a positive signal for elastin, although it is not possible to assess whether this signal is solely attributed to elastin-like nature of the vascular graft, or to elastin newly secreted by the cells. The vast majority of the stained substrate has a smooth porous structure (Supplementary Figure 2, yellow asterisk), which fits with the appearance in the bright field of the elastin-like scaffold, and therefore, the elastin signal seems to correspond mainly to the ELR-scaffold. The intense fluorescence emission of the ELR can mask the signal from newly synthetized elastin, expected to be in lower amounts. The presence of small amounts of elastin cannot, therefore, be ruled out, and positive signal within the secreted ECM (indicated with arrows in the figure) seems to corroborate this (Supplementary Figure 2).

The presence of elastin (whether it is secreted or synthetic elastin) provides the basis for an adequate elastic response to pulsatile pressure. Despite the primordial role of the elastin in ensuring appropriate mechanical function of the vessels, the expression of mature elastin in arterial tissue engineering represents still a significant challenge (Patel et al., 2006; Lee et al., 2011). We circumvent here this problem by using the ELRs as a fabrication material of the grafts. Overall, these results confirmed that the fabricated ElastinGrafts can guide cell infiltration and tissue remodeling, resulting in vascular equivalents that emulate the composition of the native vessels.

**Figure 6:**
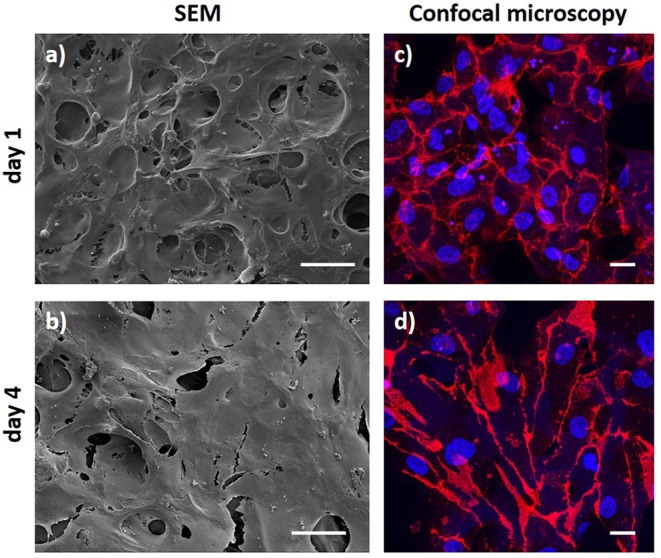
Endothelial progenitor cell layer obtained after 1 day and 4 days of culture. **(a,b)** SEM images. **(c,d)** Confocal microscopy images after CD31 staining (red) with DAPI as nuclear counterstain (blue). Scale bars: **(a,b)** 50 μm; **(c,d)** 20 μm.

The endothelium is the physiological and ideal blood-contacting surface, and additionally, it plays an important role in vascular biology, e.g., blood vessel tone, hemostasis, neutrophil recruitment and hormone trafficking (Sandoo et al., 2010; Rajendran et al., 2013). Therefore, the ability of a vascular graft to support endothelialization is crucial for the success of the implant. While transanastomotic endothelialization in humans is restricted to the immediate perianastomotic region (Zilla et al., 2007; Talacua et al., 2015), circulating EPCs are increasingly recognized as important contributors to vascular prosthesis endothelialization, making them important mediators of implant compatibility (Yu et al., 2015; Melchiorri et al., 2016). In order to evaluate the ability of the ElastinGrafts to support endothelialization, we seeded them with EPCs from human peripheral blood. Endothelial cells attached to and spread on the surface of the ELR scaffolds (Figure 6), which agrees with the presence of the integrin-mediated adhesion epitope RGD in the ELR scaffold (Table 1). After 4 days, an increase in cell density on the implant was evident, and resulted in a confluent endothelial layer. Further analysis by CD31 immunostaining confirmed the endothelialization of the ELRs grafts (Figures 6c,d).

## Conclusions

We fabricated small-caliber elastin-like vascular grafts featuring an open-porous structure while maintaining mechanical performance to withstand the arterial hemodynamic loads. Different from the clinically available prosthesis, the ElastinGrafts allow an energy-efficient transmission of pulsatile flow, and are able to match the compliance of a wide range of implantation targets. Moreover, the grafts promoted cell ingrowth and ECM deposition, which further supports their application as ready-to-use cell-free implants. Overall, these vascular grafts combine the reproducibility and off-the-shelf availability normally provided by synthetic grafts with the bioactivity, hemocompatibility and remodeling behavior of autografts, and represent a promising option for coronary artery by-pass surgery. The recombinant nature of the ELRs offers the potential to incorporate further biofunctionalities in an accurate and controlled manner, in order to tune the performance of the resulting grafts.

## Data Availability Statement

Data associated with this study is available upon request to the corresponding authors.

## Ethics Statement

The umbilical cords were kindly provided by the Department of Gynecology at the University Hospital Aachen in accordance with the human subjects' approval of the ethics committee (EK 2067) upon written informed consent.

## Author Contributions

PM and AF-C designed the study and wrote the manuscript. AF-C and FW performed the experiments. SR characterized the samples by scanning electron microscopy. SJ, JR-C, and TS-R revised the manuscript. All authors approved the final version.

### Conflict of Interest

The authors declare that the research was conducted in the absence of any commercial or financial relationships that could be construed as a potential conflict of interest.

## Supplementary Material

Supplementary Figure 1 Structure of the ElastinGraft. **(a)** PVDF-mesh. **(b–d)** Macroscopic ElastinGraft images: the PVDF-mesh is fully embedded with the ELR scaffold. **(e)** SEM image of the cross-section of the ElastinGraft wall, showing the porous structure of the ELR (colored in green) completely embedding the PVDF-mesh (colored in purple with the software MountainsMap SEM software courtesy of Digital Surf, France).

Supplementary Figure 2 Morphology of the ElastinGraft before and after cell seeding. **(a)** Microscopy images of paraffin-cuts of the ElastinGraft before seeding. The grafts present a porous structure and the wall of the pores show a smooth appearance. **(b)** Microscopy images of paraffin-cuts of the ElastinGraft 12 days after cell seeding and cultivation. Scale bars: **(a)** (i), **(b)** (i) and **(b)** (iii) 50 μm. **(a)** (ii), **(b)** (ii) and **(b)** (iv) 20 μm.

Supplementary Video 1 Ultrasound video of the ElastinGraft_eM in the simulated low (20/60 and 50/90 mmHg), normal (80/120 mmHg), and high pressure ranges (110/150 mmHg). Scale bar 5 mm. File type MP4.
