# Supplementary material for: Small Caliber Compliant Vascular Grafts Based on Elastin-Like Recombinamers for in situ Tissue Engineering
Source: Front Bioeng Biotechnol. 2019 Nov 19;7:340. doi: 10.3389/fbioe.2019.00340 (PMC6877483; doi:10.3389/fbioe.2019.00340)
Supplement: Supplementary Figure 1 — Structure of the ElastinGraft. (a) PVDF-mesh. (b–d) Macroscopic ElastinGraft images: the PVDF-mesh is fully embedded with the ELR scaffold. (e) SEM image of the cross-section of the ElastinGraft wall, showing the porous structure of the ELR (colored in green) completely embedding the PVDF-mesh (colored in purple with the software MountainsMap SEM software courtesy of Digital Surf, France). [file Data_Sheet_1.PDF]

Supplementary Material

1 Supplementary Figures

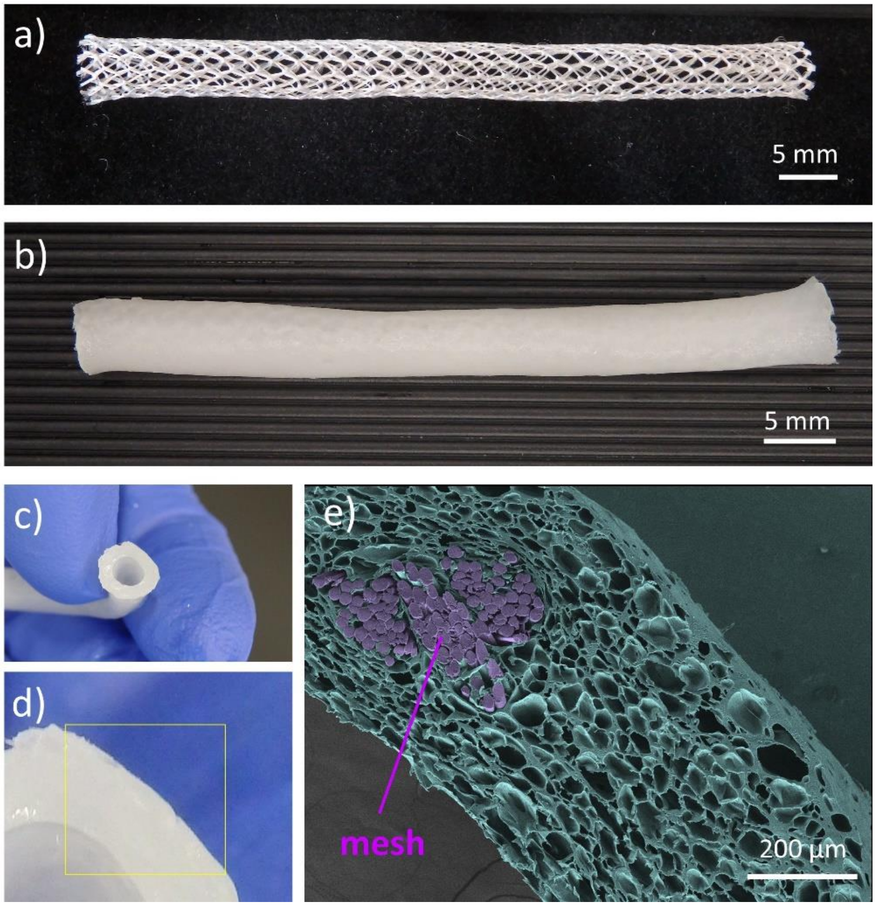

Supplementary Figure 1: Structure of the ElastinGraft. a) PVDF-mesh. b)-d) Macroscopic ElastinGraft images: the PVDF-mesh is fully embedded with the ELR scaffold. e) SEM image of the cross-section of the ElastinGraft wall, showing the porous structure of the ELR (coloured in green) completely embedding the PVDF-mesh (coloured in purple with the software MountainsMap SEM software courtesy of Digital Surf, France).

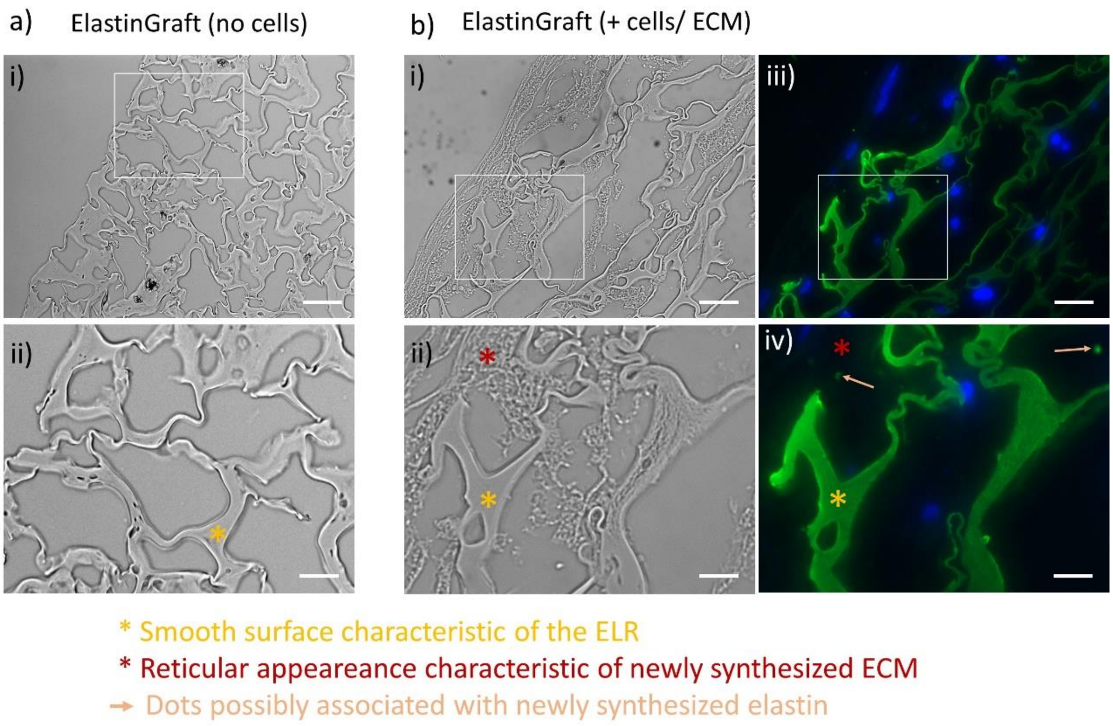

Supplementary Figure 2: Morphology of the ElastinGraft before and after cell seeding. a) Microscopy images of paraffin-cuts of the ElastinGraft before seeding. The grafts present a porous structure and the wall of the pores show a smooth appearance. b) Microscopy images of paraffin-cuts of the ElastinGraft 12 days after cell seeding and cultivation. Scale bars: a) i), b) i) and b) iii) 50 µm. a) ii), b) ii) and b) iv) 20 µm.
